# Hierarchical motor competencies and academic achievement: visual-motor integration as the key correlate for school-age children in a disadvantaged context

**DOI:** 10.3389/fpsyg.2026.1829790

**Published:** 2026-06-11

**Authors:** Xue Li

**Affiliations:** Independent Researcher, Zhengzhou, Henan, China

**Keywords:** academic achievement, cognitive demand, disadvantaged context, motor competence, school-age children, visual-motor integration

## Abstract

**Purpose:**

The relationship between motor competencies and academic achievement in children from disadvantaged backgrounds remains unclear. This study proposes a theoretical framework titled “Motor Competency–Academic Achievement Link Based on Cognitive Demand Hierarchy” to guide subsequent investigations into the association between hierarchically structured motor competencies and academic achievement among children from disadvantaged backgrounds. We hypothesize that visual-motor integration within the sensorimotor integration dimension, which requires active higher-order cognitive involvement, may exhibit a significant association with academic achievement.

**Methods:**

A cross-sectional study design was employed, with a sample of 155 children aged 6–12 years from a rural primary school in a mountainous region of Yunnan, China. Hierarchical motor competencies were assessed, including speed, hand dribbling, and visual-motor integration measured using a modified five-figure version of the Bender-Gestalt test. Academic achievement was operationalized as a composite index derived from standardized final examination scores. Hierarchical multiple regression analysis was conducted to examine the extent to which each motor competency level was uniquely associated with academic achievement, while controlling for demographic covariates.

**Results:**

Hierarchical regression analysis, controlling for grade, gender and BMI, revealed that visual-motor integration was a significant correlate of overall academic achievement (β = 0.436, *p* < 0.001). Domain-specific analyses showed that visual-motor integration significantly correlated with both mathematics (β = 0.419, *p* < 0.001) and Chinese language (β = 0.393, *p* < 0.001) performance. Basic speed measures showed a significant association with Chinese language only when grade was controlled. However, sensitivity analyses omitting grade indicated that this association was not robust and was likely confounded by developmental differences. In contrast, the correlates between visual-motor integration and academic achievement remained statistically significant across different nutritional status groups and were confirmed in multiple sensitivity analyses.

**Conclusion:**

The present findings provide preliminary evidence that, in resource-limited settings, children's visual-motor integration may be a domain-general correlate of academic achievement. In contrast, automated motor skills did not emerge as a significant correlate of academic achievement. Speed does not show an independent association after accounting for grade-related maturational differences.

## 1 Introduction

Globally, a central challenge in advancing educational equity lies in effectively supporting the developmental needs of children in disadvantaged regions. This challenge is particularly salient in the mountainous areas of Yunnan, China, where geographic isolation, limited economic resources, multi-ethnic diversity, and population mobility converge. As a result, many children in these areas are exposed to multiple environmental risks during early development, including nutritional inadequacy (Fellmeth et al., 2018), limited access to educational resources (Li et al., 2020; Liu et al., 2022), reduced emotional support due to the prevalence of boarding school arrangements (Bassett-Gunter et al., 2017; van Wyk et al., 2020), and language barriers faced by children from ethnic minority backgrounds (Xu et al., 2003). These adverse factors may impair the neurocognitive development that underpins learning abilities and hinder academic progress (Castelli et al., 2007; Du et al., 2022; Eveland-Sayers et al., 2009; Goldberg, 2022; Qiu et al., 2023; Zhang et al., 2021).

There is growing consensus that physical activity confers cognitive benefits, but the mechanisms underlying this relationship remain unclear. Developmental cognitive neuroscience suggests that the cognitive effects of motor activities depend critically on the specific neurocognitive processes they engage (Fernández-Sánchez et al., 2025; Wei et al., 2024). Embodied cognition theory posits that higher cognitive functions are grounded in sensorimotor experience and that cognition and action share common neural substrates (Craighero, 2022). Within this framework, sensorimotor integration (SMI) is conceptualized as a foundational process for higher-order cognition, referring to the efficiency with which an individual receives, processes, and integrates visual, proprioceptive, and other sensory information to plan, execute, and modify goal-directed actions (García-Madruga et al., 2016). Research suggests a connection between the processing of numbers, space, and abstract concepts and the activation of specific neural networks associated with sensorimotor experiences (Ju, 2016). Disadvantaged environments may constrain SMI development, with potential implications for the neurocognitive processes that underpin learning.

This study focuses on one specific, measurable component of SMI: visual-motor integration (VMI). VMI refers to the efficiency with which an individual coordinates visual perception and fine motor movements, a process that requires the real-time integration of visual input with motor output. As a core facet of the broader sensorimotor system, VMI has been shown to engage the prefrontal–parietal networks that also support higher-order academic skills (Wang et al., 2024).

**Figure 1:**
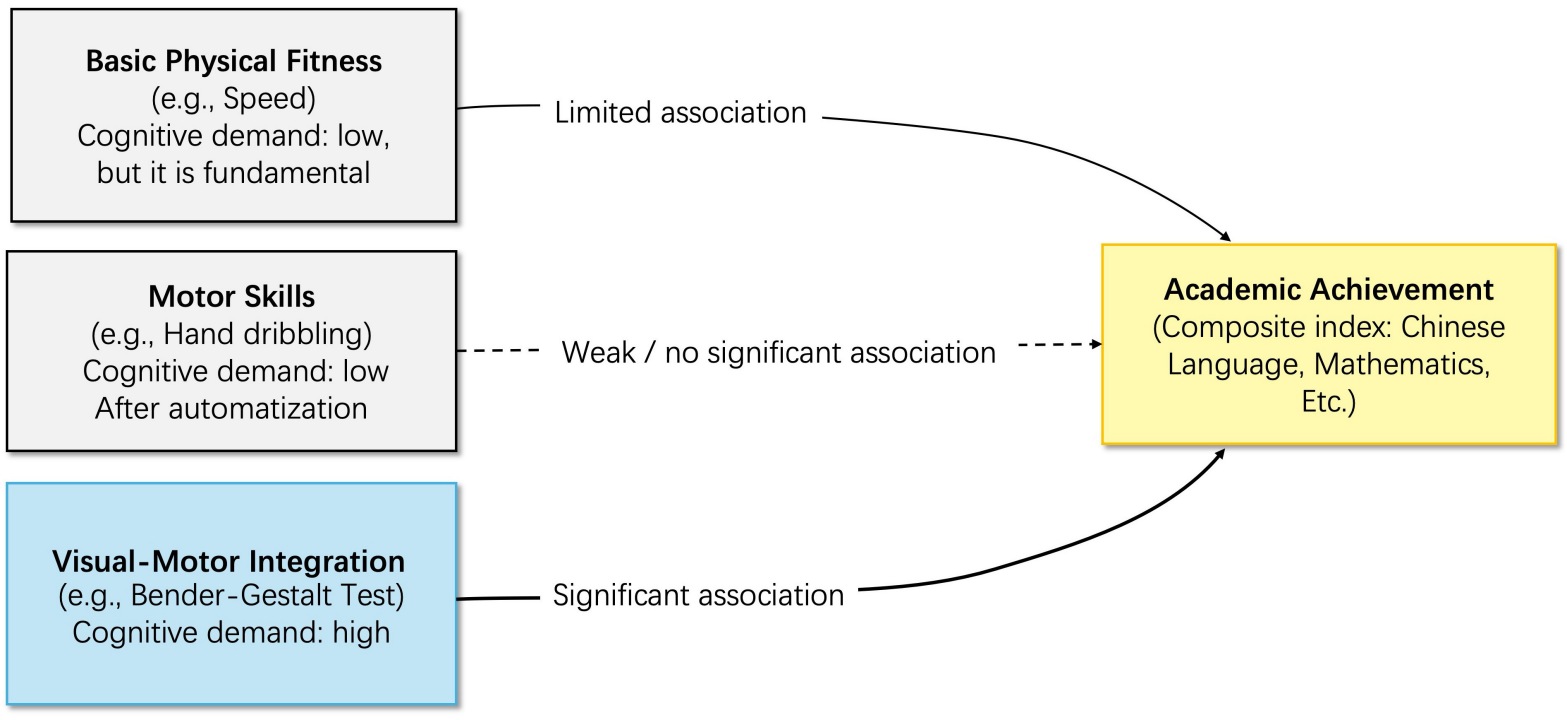
The motor competency–academic achievement link based on cognitive demand hierarchy model. All associations are based on hierarchical regression analyses controlling for grade, sex, and nutritional status (BMI).

To explore the preliminary relationship between motor competencies involving high cognitive engagement and academic achievement among children from disadvantaged backgrounds, this study proposes a theoretical model of the “Motor Competency–Academic Achievement Link Based on Cognitive Demand Hierarchy” (Figure 1). The model posits that motor competencies can be differentiated by varying cognitive demands. The most fundamental is physical fitness. Physical fitness reflects physiological capacity; although it involves low cognitive engagement, it provides foundational resources for cognitive activities (Khalaji et al., 2025). Next is motor skill, which demands higher cognitive engagement during acquisition but can become automated through training or frequent use. Once consolidated, it requires only minimal cognitive supervision (Holmes and Spence, 2004; Sattin et al., 2023). Finally, the core component of SMI is VMI. It maintains the dynamic coupling between perception and action and shares cognitive resources with learning-related processes (Silva et al., 2012).

This hierarchical distinction can also be understood through the lens of shared neural substrates. Neuroimaging studies reveal that when individuals complete complex sensorimotor tasks, the brain activates a widespread neural network. This network encompasses the prefrontal cortex and parietal regions. Interestingly, these brain areas are also precisely where higher-level learning skills are supported (Salmi et al., 2010; Stoodley and Schmahmann, 2009). In contrast, simple physical activities primarily engage subcortical structures and the cerebellum. The prefrontal cortex plays a relatively minor role in this process. Once a motor skill is mastered, it is mainly controlled by the basal ganglia and the sensorimotor cortex, at which point performing the action requires minimal cognitive effort (Torbati et al., 2024). VMI is a core component of SMI that requires individuals to coordinate perceptions and actions in real time, tasks that continuously engage the prefrontal-parietal network. This suggests that VMI and academic learning may draw upon overlapping cognitive resources (Bhandari and Badre, 2020). Accordingly, the neural overlap documented in the literature offers a theoretical rationale for why VMI would be expected to correlate with academic achievement.

In resource-limited environments, environmental pressures may exert differential effects on different levels of motor competencies. Based on this idea, we propose three hypotheses:

H1: VMI constitutes the core component of SMI. It shares neural resources with higher-order cognitive processes. Therefore, VMI may show a significant association with academic achievement.

H2: Automated motor skills are primarily controlled by subcortical circuits with minimal prefrontal involvement. Such skills may show weak or no association with academic achievement.

H3: While physical fitness also requires some cognitive engagement, this demand is relatively limited. It may show specific associations with subjects requiring rapid information processing. However, its overall degree of correlation would be weaker than that of VMI.

To empirically test these hypotheses, the three levels of motor competencies were operationalized using measures appropriate for children aged 6 to 12 years. We measured physical fitness using speed-related tasks. Two reasons led us to select speed as an indicator of basic physical fitness. First, speed ability is in a sensitive developmental phase during this age range (Van Hooren and De Ste Croix, 2020). Second, measuring speed is relatively easy to implement in resource-limited field settings. Furthermore, speed tasks requiring directional changes also reflect cognitive abilities such as information processing speed and attention switching. These cognitive abilities may be related to learning capacity (Schott and Klotzbier, 2018). Motor skills were assessed using a hand dribbling task. Finally, VMI was assessed using a modified five-figure version of the Bender-Gestalt test (B-G test), a neuropsychological assessment tool. During the test, children are asked to copy geometric shapes. This task requires them to integrate visual perception, spatial analysis, and fine motor planning in real time. Therefore, this test effectively reflects the process of cognitive–motor coupling.

## 2 Methods

### 2.1 Participants

All participants were recruited from a primary school in southern Yunnan Province, China. The school was a small-scale rural boarding institution. According to local education bureau statistics, the teacher-to-student ratio in rural primary schools was 1:15, and the boarding rate was 100%. Students from economically disadvantaged families received annual boarding subsidies of 1,250 yuan during compulsory education. These characteristics were broadly representative of rural schools in the region. An initial sample of children in grades 1 through 5 was recruited in June 2024. Inclusion criteria were age between 6 and 12 years and the absence of severe physical illness or cognitive impairment. Based on this criterion, we recruited a total of 156 children. To eliminate the impact of developmental differences across grade levels and enhance the reliability of statistical analysis, we performed a data processing step. For each physical ability metric and academic achievement, we created box plots by grade level to identify outliers. Values falling between 1.5 and 3 interquartile ranges below the first quartile or above the third quartile were classified as outliers; values beyond 3 interquartile ranges were classified as extreme values. For each flagged data point, we carefully examined the specific circumstances. For outliers in academic performance, we assessed whether they were educationally plausible. For outliers in motor performance, we determined whether the anomalous values were replicated in other tests. Based on this review, participants were assigned to one of four categories: (1) clean sample (*n* = 137), (2) outliers (*n* = 13), (3) plausible extreme values (*n* = 5), and (4) special case (*n* = 1). The main analyses combined groups 1, 2, and 3, resulting in an analytical sample of 155 children. We did this to maximize the use of available data while ensuring statistical reliability. The influence of this classification decision was examined in sensitivity analyses.

### 2.2 Variables assessment

#### 2.2.1 Academic achievement

Academic achievement data were obtained from final examination scores for the spring semester of 2024. Examination papers were developed centrally by the Yunnan Provincial Department of Education. The subjects assessed varied by grade level. Chinese language and mathematics were administered in grades 1 through 5. English was additionally administered in grades 3 through 5, moral education in grades 3 and 5, and science in grade 4. Chinese and mathematics were compulsory core subjects across all grades, whereas English was introduced beginning in grade 3. Other subjects were typically assessed through spot checks and are considered non-core, unlike the practice in many urban schools.

To ensure comparability across grades and subjects, raw scores were converted to within-grade, within-subject z-scores. Cronbach's alpha was then calculated for all students' common exam subjects within each grade. The results indicated excellent reliability, with most grades achieving alpha coefficients exceeding 0.8. The second-grade coefficient of 0.77 was also acceptable. To further assess data quality, item–total correlations were examined. Both mathematics (*r* = 0.63) and Chinese (*r* = 0.63) exceeded the recommended threshold of 0.5, indicating acceptable internal consistency. Based on this evidence, the standardized scores were considered adequate for subsequent analyses. For each child, a composite academic index was computed as the mean of the z scores across all available subjects, providing an overall indicator of academic achievement. Although academic scores were standardized within each grade, we retained grade as a covariate because motor performance measures were not grade-standardized and are developmentally sensitive. Grade served as a proxy for maturational stage, controlling for developmental differences that could otherwise confound motor–academic associations. This conservative approach may have overcontrolled variance.

#### 2.2.2 Motor competencies

Speed and hand dribbling tests were conducted using standardized procedures during physical education lessons across all grades. Each participant was afforded two attempts per test, with the highest score recorded as the result. The B-G test was administered during indoor classroom sessions.

##### 2.2.2.1 Speed

30-m Sprint: Each participant assumed a standing start position, facing forward, and sprinted 30 meters at maximum speed. Timing began when the participant-initiated movement and stopped upon crossing the 30-m finish line.

3 × 10-m Shuttle Run: The test area consisted of two parallel lines spaced 10 meters apart. At each end, a semicircle with a radius of 0.5 meters was drawn, and a small ball (Ball A and Ball B) was placed at the center of each semicircle. The participant started from a standing position at the point where the starting line met the semicircle. Upon the tester's verbal command, the participant sprinted at full speed to Ball B, ran around it, turned back to Ball A, ran around it, and then sprinted past Ball B to complete the test. Timing began when the participant started running and stopped when the participant crossed the line at Ball B.

To reduce multicollinearity and form a theoretical construct, the best score from each of the two tests was standardized using the following formula: standardized value = (value – mean) / standard deviation. After standardization, the average of the two standardized scores was calculated and used as the composite speed score.

##### 2.2.2.2 Hand dribbling

The hand dribbling test was conducted on a 10-meter course with marker poles placed every 2.5 meters. Participants started from the starting point, dribbled the ball with one hand, and weaved through three marker poles before reaching the finish line. They were instructed to stay as close as possible to each pole while weaving and to complete the task in the shortest possible time. Each participant performed the test using both their dominant and non-dominant hands, following an alternating order: dominant hand, non-dominant hand, dominant hand, non-dominant hand, for a total of four attempts. If the ball went out of the participant's control during a trial, the attempt was restarted. Children aged 6 to 9 years completed the test using a football; those aged 10 years and above used a basketball.

To examine whether the hand dribbling task captured automatized performance, paired-sample *t*-tests were conducted comparing the first and second attempts for each hand. For the dominant hand, completion time was significantly longer in the second attempt (*M* = 7.21s, *SD* = 2.36) compared to the first (*M* = 6.95s, *SD* = 2.51), *t*_(154)_ = −2.174, *p* = 0.031, Cohen's *d* = 0.18. Similarly, for the non-dominant hand, the second attempt (*M* = 7.37s, *SD* = 3.76) was significantly slower than the first (*M* = 6.61s, *SD* = 2.24), *t*_(154)_ = −2.777, *p* = 0.006, Cohen's *d* = 0.22. The significant increases in completion time, coupled with the small effect sizes, suggested a mild fatigue effect rather than learning. Importantly, no improvement was observed across trials, indicating that performance had already reached a plateau. However, we acknowledge that this is only an indirect indicator of automation. It remains possible that the task still requires some cognitive engagement, or that our measurement was not sensitive enough to detect learning effects. Therefore, while we interpret hand dribbling as reflecting a well-practiced skill, we recognize that definitive classification would require direct measurement of cognitive load.

Consistent with the speed test processing procedure, the results from the hand dribbling test for the dominant and non-dominant hands were standardized and combined to form a dribbling composite score.

##### 2.2.2.3 Visual-motor integration

**Figure 2:**
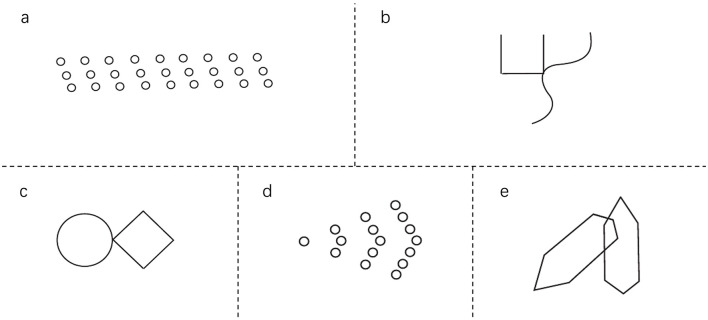
The five-figure abbreviated version of the Bender-Gestalt test used in this study. **(a)** Three rows of nine circles, each row tilted right; **(b)** square missing top side with wavy line at lower-right; **(c)** circle overlapping a diamond; **(d)** right arrow made of 1,3,5,7 small circles; **(e)** two overlapping hexagonal diamonds.

VMI was assessed using a modified version of the Bender-Gestalt test. This modified version consisted of five geometric figures reduced from the original nine due to time and resource constraints (Figure 2). As an instrument for measuring VMI, this test captured the efficiency with which individuals coordinate visual perception and fine motor output. To obtain a more direct understanding of children's VMI, a scoring system revised and supplemented by Russian scholars was adopted (Lovi and Belopolsky, 2008). This system was selected because it addressed the practical challenges associated with storing and interpreting collected data in the form of images or detailed protocols. It also overcame limitations of the original normative system, which did not capture all variations that could meaningfully influence result interpretation. The system has demonstrated satisfactory reliability and validity.

To assess the content validity of this modified version, we invited five experts in psychology and education to evaluate it. Each expert used a four-point scale to rate the association between these five graphics and the VMI concept. The results showed that the content validity indices for the five graphics at the item level ranged from 0.8 to 1.0, with four graphics achieving the maximum score of 1.0. The content validity index at the scale level, calculated using the average method, was 0.96. This indicated that the modified version possessed good content validity. Within the study sample, the internal consistency reliability of this modified version was also acceptable. Its Cronbach's alpha coefficient was 0.725, with corrected item-total correlations ranging from 0.43 to 0.58. We also conducted exploratory factor analysis. The KMO value was 0.767, and Bartlett's sphericity test yielded significant results. Principal component analysis extracted one factor with an eigenvalue greater than 1, explaining 48.32% of the total variance. Factor loadings for each graphic ranged from 0.636 to 0.78, all exceeding the 0.5 benchmark. This indicated each graphic made a substantive contribution to the measurement of VMI. To assess scoring consistency, a random subset of 100 B-G test protocols (approximately 65% of the sample) was independently scored by a second trained researcher who was blind to all child characteristics and the original scores. Both scorers followed the detailed scoring criteria provided in Supplementary Table S1. The intraclass correlation coefficient (ICC) was computed using a two-way mixed effects model for absolute agreement. The single-measures ICC was 0.946 (95% CI: 0.921–0.964, *p* < 0.001), indicating excellent inter-rater reliability.

Nonetheless, we must acknowledge a significant limitation in this study's methodology for assessing VMI. Although content validity, construct validity, internal consistency, and inter-rater reliability all reached acceptable levels, we did not use the full nine-figure version as a criterion to test the criterion-related validity of this modified version. Therefore, while this five-figure modified version could measure certain aspects of VMI, scores derived from it might not be fully equivalent to those from the full Bender-Gestalt test. We must exercise caution when interpreting the research findings. Consequently, our findings should be interpreted as preliminary, and direct comparisons with studies using the full Bender-Gestalt test are not warranted. Future studies should therefore employ the full nine-figure Bender-Gestalt test or other well-validated VMI measures to confirm and extend our findings.

The children were informed that the test was not timed, but because it took place during regular 45-min class sessions, they may have felt an implicit time constraint that could have affected their performance. The test was administered on blank white A4 paper, and no auxiliary tools were permitted. The five geometric figures were presented to each participant in sequence for copying. After completion, each figure was scored individually according to the assessment scale, and the total score across all five figures was recorded. Scoring criteria for each figure were based on fidelity to the original shape, spatial proportion, accuracy of angles, and quality of line connections. Total scores ranged from 5 to 25. Detailed scoring guidelines are provided in Supplementary Table S1. For unified analysis, this total score was subsequently converted to a standardized score.

##### 2.2.2.4 Anthropometric variables

Height and weight of the children were measured using scales and height rulers on the school playground. Body mass index (BMI) was calculated using the formula weight (kg) / height^2^ (m^2^) to obtain raw BMI values. To control for the effects of age and gender, we used linear regression to obtain the unstandardized residuals of BMI regressed on age and gender. These residuals were treated as age-and-gender-standardized z scores (z-BMI) and were included in subsequent analyses. Information on grade and gender was also included.

### 2.3 Statistical analysis

#### 2.3.1 Data processing

To address deviations from normality prior to parametric analysis, variables with non-normal distributions were transformed using the natural logarithm. This transformation was applied only within specific grades where adjustment was needed, specifically the 30-m sprint test for Grade 5 children and the hand dribbling test for Grade 3 children. Validation analyses confirmed that these localized transformations did not alter the direction or statistical significance of the relationships between the transformed variables and academic achievement. For the small proportion of randomly missing values (less than 5%) in the regression models, multiple imputation using chained equations was performed (*m* = 5), and the imputed results were pooled. All statistical analyses were conducted using IBM SPSS Statistics (Version 29.0.1.0). The significance level was set at α = 0.05 (two-tailed).

#### 2.3.2 Descriptive statistics for participants and variables

For continuous variables, descriptive statistics were calculated and reported as means and standard deviations. Categorical variables were summarized using frequencies and percentages.

#### 2.3.3 Relationship between academic achievement and all other measured variables

The BMI, academic achievement index, speed composite score, dribbling composite score, Chinese language, mathematics, 30-m sprint test, 3 × 10-m shuttle run test, hand dribbling task (dominant hand; non-dominant hand), and B-G test scores were retained for analysis. Pearson correlation coefficients were calculated to examine bivariate associations among these variables.

#### 2.3.4 Primary analysis model

Hierarchical multiple regression analysis was conducted, with the composite academic achievement index as the dependent variable. In the first step (Model 1), control variables including grade, gender, and z-BMI were entered. In the second step (Model 2), the three motor variables, B-G test score, speed composite score, and dribbling composite score were entered simultaneously. The variance explained by each model (*R*^2^), the change in *R*^2^ (Δ*R*^2^) and its significance, as well as standardized regression coefficients (β), standard errors, 95% confidence intervals, and *p-values* for all associated factors were reported. Collinearity diagnostics were assessed using variance inflation factor (VIF). VIF values for all associated factors in all models were below 10, indicating that multicollinearity was within an acceptable range.

#### 2.3.5 Exploratory analysis

To investigate differences in the relationship between various subjects and motor competencies, we repeated the hierarchical regression analyses using mathematics z-scores and Chinese language z-scores as dependent variables.

To examine whether nutritional status moderated the relationship between motor competencies and academic achievement, we divided the sample into two groups: children with malnutrition (*n* = 26) and children without malnutrition (*n* = 129). Hierarchical regression analyses were then repeated separately for each group. Finally, we employed Fisher's z-test to compare whether there was a significant difference in the strength of association between B-G test scores and academic performance between the two groups.

#### 2.3.6 Sensitivity analysis

Two sensitivity analyses were conducted. First, the main regression models were repeated using the clean sample (*n* = 137) and the full sample (*n* = 156). Second, academic achievement scores were recalculated using only the z scores for Chinese language and mathematics, and the regression analyses were performed again. Additionally, the primary hierarchical regression model (*n* = 155) was rerun without the grade variables to test the stability of the results across alternative model specifications.

## 3 Results

### 3.1 Descriptive statistics for participants and variables

Table 1 shows the final analytical sample for this study consisted of 155 children (56.1% boys), with a mean age of 9.6 years (*SD* =1.76, range 6–12 years). In terms of nutritional status, 16.7% of the children had BMI values falling within the range of the BMI-for-age screening cutoff for wasting, as specified in the screening standard for malnutrition in school-age children and adolescents (WS/T 456-2014) for Chinese children and adolescents (National Health Commission of the People's Republic of China, 2014).

**Table 1:** Descriptive statistics and distribution of key variables (*N* = 155).

Variable	*M*	*SD*
Demographic characteristics		
Age (years)	9.6	1.756
Gender (% male)	56.1	
Wasting status (% wasted)	16.7	
BMI	16.046	2.097
BMI (*z*-score)	0.000	1.000
Motor competence (*z*-score)		
Speed composite^a^	0.000	0.854
Dribbling composite^b^	0.000	0.989
B-G test^c^	0.003	1.002
Academic achievement (within-grade *z*-scores)		
Chinese language	0.003	0.987
Mathematics	−0.004	0.986
Academic index	0.003	0.893

^a^Speed composite is the average of z-standardized scores from the 30-m sprint and 3 × 10-m shuttle run. Raw means (SD) were: 30-m sprint = 6.17(0.65) s, shuttle run = 10.54 (0.72) s. ^b^Dribbling composite is the average of z-standardized scores from dominant and non-dominant hand dribbling. Raw means (SD) were: dominant hand = 6.55 (2.11) s, non-dominant hand = 6.37 (2.05) s. ^c^Modified Bender-Gestalt Test scores range from 5 to 25, with higher scores indicating better VMI. Raw means (SD) were: 13.52 (3.14).

### 3.2 Relationship between academic achievement and all other measured variables

The 30-m sprint and 3 × 10-m shuttle run were moderately correlated (*r* = 0.46, *p* < 0.01), supporting their combination into a composite speed score to reduce multicollinearity in subsequent analyses. Similarly, dominant and non-dominant hand dribbling performances were very highly correlated (*r* = 0.956, *p* < 0.01), justifying the creation of a single dribbling composite.

The modified B-G test demonstrated significant positive correlations with all learning indicators: Chinese language (*r* = 0.348, *p* < 0.01), mathematics (*r* = 0.336, *p* < 0.01), and the composite academic index (*r* = 0.375, *p* < 0.01). These moderate effect sizes indicated that children with better VMI achieved higher academic achievement. In contrast, the speed composite score (*r* = −0.08, *p* = 0.32) and dribbling composite score (*r* = −0.014, *p* = 0.86) showed no significant correlations with any academic achievement measure. The coefficients were small and negative, indicating weak and statistically negligible bivariate relationships. Although non-significant, the negative correlations were consistent with expectations. The speed composite score showed a moderate positive correlation with the dribbling composite score. Both the speed composite score (*r* = −0.207, *p* < 0.01) and the dribbling composite score (*r* = −0.259, *p* < 0.01) exhibited significant negative correlations with the B-G test.

These correlations also indicated that children with higher VMI scores tended to perform better on speed and hand dribbling tasks. BMI showed no significant correlation with any motor or academic variable (see Supplementary Table S2).

### 3.3 Hierarchical regression analysis: differential associations of motor competencies with academic achievement

Table 2 shows that after controlling for grade, gender, and z-BMI, the demographic variables alone explained minimal variance in composite academic achievement (*R*^2^ = 0.4%, adjusted *R*^2^ = −0.037, *p* = 0.965). When the three motor competency variables were added to the model, the explanatory power increased substantially, accounting for an additional 19.3% of the variance (Δ*R* = 0.193, *p* < 0.001). Among the motor variables, only the B-G test score showed a significant independent association (β = 0.436, *p* < 0.001), indicating that children with better VMI tended to have higher academic scores. The speed composite score also reached statistical significance (β = −0.345, *p* = 0.02). In contrast, the dribbling composite score showed no significant association (β = −0.002, *p* = 0.992). All motor competence variables were measured in seconds, with lower values indicating faster/better performance. Therefore, negative regression coefficients indicated that children with faster completion times achieved higher academic scores.

**Table 2:** Hierarchical regression analysis: associations with composite academic achievement (*N* = 155).

Variable	Model 1		Model 2		Collinearity
	*B (SE)*	β	*B (SE)*	β	VIF
Step 1: Control variables					
Grade 1	−0.012 (0.208)	−0.006	0.987 (0.415)	0.459^*^	6.735
Grade 2	0.019 (0.250)	0.007	0.790 (0.308)	0.291^*^	2.321
Grade 3	−0.011 (0.257)	−0.004	0.445 (0.361)	0.160	3.057
Grade 4	−0.019 (0.204)	−0.009	0.597 (0.269)	0.293^*^	3.159
Gender (male)	−0.089 (0.149)	−0.048	−0.114 (0.156)	−0.063	1.364
BMI (*z*-score)	0.017 (0.037)	0.038	0.033 (0.033)	0.077	1.083
Step 2: Motor competencies					
Speed composite			−0.360 (0.154)	−0.345^*^	3.899
Dribbling composite			−0.002 (0.144)	−0.002	4.570
B-G test			0.388 (0.073)	0.436^***^	1.199
Model summary					
*R^2^*		0.004		0.197	
Adjusted *R^2^*		−0.037		0.147	
*ΔR^2^*		0.004		0.193	
*F* for *ΔR^2^*		0.091		11.625^***^	

^*^p < 0.05, ^***^p < 0.001.

Among the control variables, several grade indicators became significant in Model 2 (Grade 1: β = 0.459, *p* = 0.018; Grade 2: β = 0.291, *p* = 0.034; Grade 4: β = 0.293, *p* = 0.043), indicating that after accounting for motor competencies, grade-related differences in academic achievement emerged. Gender and z-BMI remained non-significant throughout.

### 3.4 Domain-specific analyses: differential associations between motor competencies and mathematics vs. Chinese achievement

Table 3 shows that for mathematics performance, the modified B-G test was the only statistically significant correlate (β = 0.419, *p* < 0.001), whereas the speed composite score was not significant (β = −0.210, *p* = 0.162). In contrast, for Chinese language performance, both the B-G test (β = 0.393, *p* < 0.001) and the speed composite score (β = −0.428, *p* = 0.004) emerged as significant correlates.

**Table 3:** Domain-specific regression analyses for mathematics and Chinese achievement (*N* = 155).

Variable	Mathematics (z-score)		Chinese (*z*-score)	
	Model 1 (β)	Model 2 (β)	Model 1 (β)	Model 2 (β)
Step 1: Control variables				
Grade 1	−0.003	0.292	−0.009	0.528
Grade 2	−0.003	0.203	0.01	0.323
Grade 3	−0.023	0.103	−0.01	0.186
Grade 4	−0.027	0.183	−0.011	0.338
Gender (male)	0.06	0.082	−0.107	−0.151
BMI (*z*-score)	0.092	0.126	0.05	0.09
Step 2: Motor competencies				
Speed composite		−0.210		−0.428^**^
Dribbling composite		0.058		−0.018
B-G test		0.419^***^		0.393^***^
Model summary				
*R^2^*	0.012	0.172	0.014	0.194
Adjusted *R^2^*	−0.028	0.12	−0.026	0.144
*ΔR^2^*	0.012	0.16	0.014	0.181
*F* for *ΔR^2^*	0.295	9.322^**^	0.343	10.839^***^

^**^p < 0.01, ^***^p < 0.001.

Although the speed composite score was significantly associated with overall academic achievement (Table 2), the domain-specific analyses (Table 3) revealed that this association was driven entirely by Chinese language performance (β = −0.428, *p* = 0.004). The association with mathematics was not statistically significant (β = −0.210, *p* = 0.162). This pattern suggested that the contribution of the speed composite score to the composite academic achievement may have come from its link to Chinese language rather than mathematics.

Given that the two speed tests might impose different cognitive demands, a *post-hoc* analysis was conducted by entering the 30 m sprint and the shuttle run separately into the regression model for Chinese language achievement.

After controlling for grade, gender, and z-BMI, the B-G test remained a significant correlate in both models (Model 1: β = 0.379, Model 2: β = 0.374, *p* < 0.001). In Model 2, the shuttle run emerged as a significant negative correlate (β = −0.246, *p* = 0.009), indicating that faster shuttle run performance was associated with higher Chinese language scores. In contrast, the 30 m sprint did not reach significance (β = −0.017, *p* = 0.888). This suggested that, within the grade-controlled model, the association between speed and Chinese language proficiency was primarily reflected in the shuttle run task, potentially because the shuttle run demands higher cognitive requirements than the 30-meter sprint. Multicollinearity was within acceptable limits (all VIFs < 3), confirming the stability of these estimates (Table 4). If this pattern were replicated in future studies, it would complicate the simple three-tier hierarchy proposed in theoretical models. Whilst we categorized shuttle running as a speed-based activity, it might require some degree of higher cognitive involvement, suggesting that the boundaries between the proposed tiers may be more fluid than initially conceptualized. This point was further elaborated in the Discussion. It should be noted, however, that this finding was conditional on grade being included as a covariate; when grade was removed from the model (Section 3.6), the overall speed effect was no longer significant.

**Table 4:** Hierarchical regression analysis: associations of Chinese achievement with 30-m sprint and shuttle run (*n* = 155).

Variable	Model 1		Model 2		Collinearity
	*B (SE)*	β	*B (SE)*	β	VIF
Grade (continuous)	−0.108 (0.080)	−0.167	−0.151 (0.080)	−0.234	2.764
Gender (male)	−0.087 (0.153)	−0.044	−0.258 (0.164)	−0.13	1.234
BMI	0.038 (0.037)	0.079	0.049 (0.036)	0.102	1.058
30-m sprint	−0.067 (0.121)	−0.068	−0.017 (0.120)	−0.017	2.68
B-G test	0.373 (0.080)	0.379^***^	0.368 (0.078)	0.374^***^	1.141
3 × 10-m shuttle run			−0.243 (0.092)	−0.246^**^	1.57
Model summary					
*R^2^*		0.142		0.181	
Adjusted *R^2^*		0.113		0.147	
*ΔR^2^*		0.142		0.039	
*F* for *ΔR^2^*		4.934^***^		6.959^**^	

All continuous variables (including BMI, B-G test, and speed measures) were standardized to z-scores; thus, coefficients are comparable. ^**^p < 0.01, ^***^p < 0.001.

**Figure 3:**
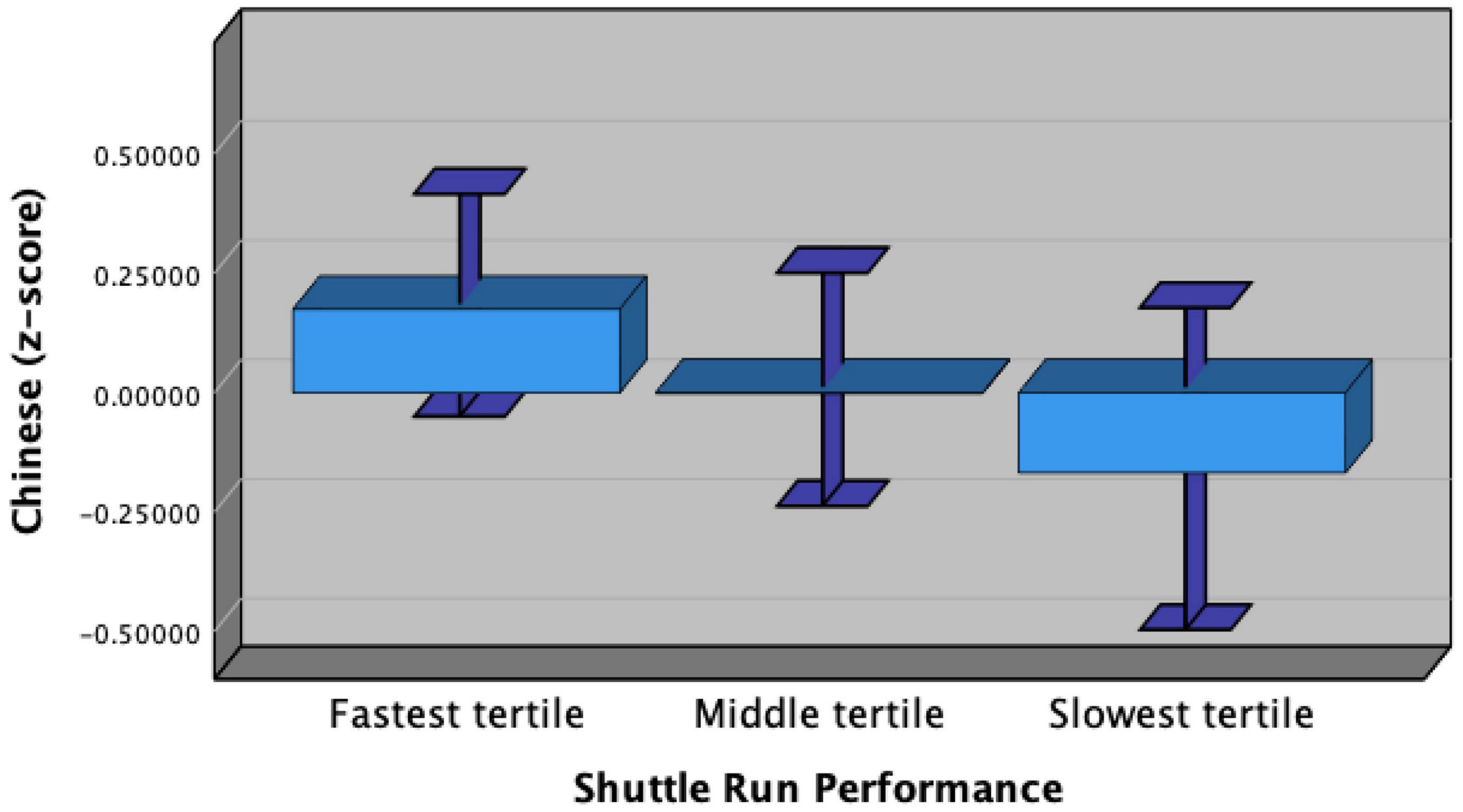
Chinese language achievements by shuttle run performance tertiles.

To present these findings more intuitively, we divided participants into three groups based on their shuttle run times: fast, medium, and slow. As shown in Figure 3, Chinese language scores followed a clear gradient pattern. The group with the fastest times had the highest average score (*M* = 0.175), followed by the middle group (*M* = −0.001), while the slowest group had the lowest average score (*M* = −0.167).

### 3.5 Exploratory analyses: nutritional status

As shown in Table 5, the modified B-G test remained a significant correlate of academic achievement in both groups (malnourished: *n* = 26, β = 0.586, *p* = 0.027; non-malnourished: *n* = 129, β = 0.419, *p* < 0.001). Fisher's *z*-test comparing the two associations yielded *z* = 0.39, *p* = 0.697, indicating no statistically significant difference.

**Table 5:** Group-specific hierarchical regression analyses: associations of academic performance by nutritional status.

Variable	Malnourished group (***n*** = 26)		Non-malnourished group (***n*** = 129)		Group comparison
	Model 1 (β)	Model 2 (β)	Model 1 (β)	Model 2 (β)	Fisher's *z*
Step 1: Control variables					
Grade 1	−0.004	0.592	−0.027	0.437	
Grade 2	0.154	0.265	−0.023	0.258	
Grade 3	0.14	0.071	−0.033	0.153	
Grade 4	0.333	0.608	−0.053	0.242	
Gender (male)	−0.175	−0.045	−0.037	−0.089	
BMI (*z*-score)	−0.103	0.014	−0.03	0.074	
Step 2: Motor competencies					
Speed composite		−0.363		−0.34^*^	
Dribbling composite		−0.335		0.026	
B-G test		0.586^*^		0.419^***^	0.39
Model summary					
*R^2^*	0.1	0.387	0.004	0.188	
Adjusted *R^2^*	−0.185	0.042	−0.045	0.127	
*ΔR^2^*	0.1	0.287	0.004	0.185	
*F* for *ΔR^2^*	0.35	2.5	0.073	9.024^***^	

The malnourished subgroup (*n* = 26) is small; interpret with caution. ^*^p < 0.05, ^***^p < 0.001.

In contrast, the speed composite score showed no significant association with academic achievement in the malnourished group (β = −0.363, *p* = 0.399) but was significantly associated in the non-malnourished group (β = −0.340, *p* = 0.031). No significant association was observed for the dribbling composite score in either the malnourished group (β = −0.335, *p* = 0.397) or the non-malnourished group (β = 0.026, *p* = 0.888).

### 3.6 Sensitivity analysis results

To assess the robustness of the main findings, the primary regression analysis was repeated under three conditions. First, the analysis was conducted using only the clean sample (*n* =137). After excluding all marked outliers, the B-G test remained a significant correlate (β = 0.384, *p* < 0.001), as did the speed composite score (β = −0.333, *p* = 0.039). The dribbling composite score was not significant (β = −0.159, *p* = 0.366). Second, the analysis was repeated using the full sample, which included one special case retained based on professional judgment (*n* =156). The results remained largely unchanged: the B-G test (β = 0.436, *p* < 0.001) and the speed composite score (β = −0.345, *p* = 0.019) were significant correlates, whereas the dribbling composite score was not (β = −0.008, *p* = 0.960). Third, academic achievement was recalculated using only the core subjects (Chinese language and mathematics) rather than the composite index that included all subjects. The B-G test remained significant (β = 0.437, *p* < 0.001), and the speed composite score showed a similar trend (β = −0.343, *p* = 0.039). The pattern of results remained consistent across all three sensitivity analyses, supporting the robustness of the study findings. Further details are available in Supplementary Table S3.

To examine whether the main findings were robust to the removal of grade, we re-ran the primary hierarchical regression without the grade dummy variables. The results are shown in Supplementary Table S4. In this model, the B-G test remained a significant correlate (β = 0.405, *p* = 0.001), whereas the speed composite score was no longer significant (β = −0.046, *p* = 0.591). The dribbling composite remained non-significant (β = 0.119, *p* = 0.155). This indicates that the speed-achievement association observed in the primary model (Section 3.3) was largely attributable to grade-related developmental differences. Therefore, the domain-specific speed findings (Section 3.4) should be interpreted with the understanding that the overall speed effect was not robust to alternative model specifications. In contrast, the VMI effect was stable across models.

## 4 Discussion

This study employed the Motor Competency–Academic Achievement Link Based on Cognitive Demand Hierarchy model to preliminarily shed light on the relationship between motor competency and academic achievement among children in resource-scarce mountainous regions of China. Within this model, VMI serves as the core component of SMI, requiring active engagement of higher-order cognitive processes. We found that VMI exhibited a significant correlation with academic performance. In contrast, motor skills that had become automated did not show such an association with academic achievement. Furthermore, in grade-controlled analyses, the speed metric demonstrated a specific correlation only with Chinese language subject scores; however, this association was not robust when grade was omitted from the model. These findings provide preliminary insights into the relationship between different levels of motor skills and academic achievement among children in disadvantaged contexts.

The findings demonstrated that VMI, as assessed by our modified five-figure B-G test, emerged as a significant correlate of composite academic achievement. This result aligns with the shared neural substrate hypothesis and is consistent with H1. Prior neuroimaging studies have shown that such visuomotor copying tasks activate prefrontal and parietal brain regions (Ogawa and Inui, 2009; Wang et al., 2024), which are also involved in reading comprehension and mathematical problem-solving (Hawes et al., 2019; Wang et al., 2020). Building on this existing evidence, we speculate that the correlation between VMI and academic achievement may reflect overlapping neural resources. However, because we did not directly measure brain activity, this interpretation remains speculative.

This speculation is consistent with a substantial body of behavioral research. Prior studies have demonstrated that VMI is closely associated with children's school readiness and academic performance (Chan, 2000; van Wyk et al., 2020). Our correlational findings are consistent with this perspective: we discovered significant correlations between VMI and both Chinese language and mathematics scores, with no evidence of subject-specific effects. Research indicates that VMI is independently and significantly associated with children's performance in mathematics, written expression, and language arts (García-Madruga et al., 2016; Pereira et al., 2011; Sulik et al., 2018; Zhao et al., 2024). For children learning Chinese, VMI explains a portion of the unique variance in reading fluency even after accounting for the influence of traditional cognitive skills like phonological awareness (Zhao et al., 2024). Taken together, these findings suggest that VMI is a domain-general correlate of academic achievement in this disadvantaged context.

The present findings also revealed that hand dribbling performance did not emerge as a significant correlate of academic achievement, a result generally consistent with H2. Our interpretation of this result is that the hand dribbling task used in this study primarily reflects a motor skill that has become highly proficient and ingrained. Once a motor skill is automated, its demand on cognitive resources is significantly reduced (Haith and Krakauer, 2018). Performance differences observed in executing such skills mainly reflect the level of practice proficiency rather than the amount of cognitive resources mobilized in real time (Chu, 2020). However, in this study we cannot rule out the possibility that the hand dribbling task still requires a certain degree of cognitive engagement. Future research could employ more rigorous testing to disentangle automated performance from that involving cognitive engagement. Although this interpretation remains to be confirmed, the finding that no association exists between hand dribbling and academic performance nonetheless supports the fundamental logic of our proposed theoretical model. Specifically, motor activities that demand sustained higher-order cognitive engagement and continuous coordination are more likely to share cognitive resources with those required for academic learning. This pattern reinforces a core premise of the model: it is not the motor skill itself that is associated with academic achievement, but rather the cognitive demands imposed during task execution.

In the grade-controlled model, the negative regression coefficient for the composite speed score indicated that faster times were associated with better performance. Specifically, the shuttle run showed a stronger individual association with Chinese language achievement than did the 30-m sprint. This pattern is partially consistent with H3. The key difference lies in the nature of the tasks. Unlike straight line sprinting, the shuttle run requires children to repeatedly change direction, engaging not only basic speed but also attentional shifting, rapid decision making, and motor planning. This requirement may therefore reflect differences in the cognitive processing demands across academic subjects. The specific association observed between speed measures and Chinese language scores may be explained by the nature of the tasks themselves (Lang et al., 2018). Research has demonstrated that a core component of reading fluency is the rapid and accurate recognition of visual words, a process that has been associated with neural processing efficiency (Cnudde et al., 2021). Thus, faster children might also perform better on Chinese language tasks requiring quick retrieval. However, because the speed effect was not robust when grade was removed (Section 3.6), this interpretation is speculative. In contrast, mathematics learning relies more heavily on information integration and logical reasoning, abilities that share greater overlap with the cognitive components involved in VMI. This distinction may explain why speed exhibited only a limited association with mathematics achievement in the present study (Nouwens et al., 2021).

It is noteworthy that the two speed tasks exhibited distinct result patterns in the study. This phenomenon offers an important implication for the theoretical model we propose. While physical fitness may correlate with academic performance, only the shuttle run demonstrated a significant association in our study, whereas the 30-meter sprint did not. In the correlation analysis, the two tasks themselves showed a significant moderate correlation (*r* = 0.46). This finding suggests that the levels delineated in our model are not mutually exclusive. Each specific motor task may exist along a continuum of cognitive engagement. Some tasks may also utilize abilities belonging to higher-order cognitive processes. Future models should consider this fluidity. Viewing cognitive demands as a continuous dimension, where each motor task occupies a specific position along this scale, may be more appropriate than treating them as distinct categories with clear boundaries.

However, a sensitivity analysis that omitted grade from the regression model revealed that the overall speed–achievement association was no longer significant (β = −0.043, *p* = 0.615). This indicates that the apparent speed effect observed in the grade-controlled model is largely attributable to grade-related developmental differences. Older children tend to be faster and also achieve higher scores. The effect is not due to an independent contribution of speed *per se*. Consequently, these speed related findings should be interpreted with caution and viewed as hypothesis generating rather than conclusive.

Notably, the non-robustness of the speed effect does not undermine our theoretical model; rather, it reinforces a key premise of the hierarchy. Motor tasks that require sustained higher-order cognitive engagement (i.e., VMI) are more likely to share cognitive resources with academic learning than are basic fitness tasks that are heavily influenced by maturational factors.

The sample in this study was distinctive, with a prevalence of wasting reaching 16.7%. This figure stands in contrast to broader global trends in childhood physical development and clearly reflects the level of nutritional risk faced by children in this mountainous region. This unique context provides an opportunity to examine the relationship between motor competencies and academic achievement under conditions of environmental adversity.

Given the small malnourished group (*n* = 26), we explored whether nutritional status moderated the observed associations (see Results 3.5). VMI remained significantly associated with academic achievement in both groups, with no evidence of a differential association (Fisher's *z* = 0.39, *p* = 0.697). This tentatively suggests that the VMI–academic achievement link may be robust to concurrent nutritional status. One possible explanation is that a single-time BMI measure does not fully capture cumulative nutritional effects on neurodevelopment (Castelli et al., 2007; Du et al., 2022). In contrast, the speed composite score was associated with academic achievement only in the non-malnourished group. A speculative interpretation is that fundamental physical abilities may serve as a physiological foundation for cognitive capacity. When nutritional intake is inadequate, the body may enter an energy-conserving state, potentially limiting baseline physical performance and hindering its relationship with cognitive processes. Conversely, when nutritional needs are adequately met, faster reaction times may be associated with cognitive advantages associated with academic achievement (Fanjiang and Kleinman, 2007). However, given the exploratory nature of these analyses and the small subgroup size, this pattern requires replication in larger samples.

An alternative interpretation is that under adverse environmental conditions, individual differences in children's neuropsychological functioning may become more pronounced, emerging as a key factor shaping divergent developmental outcomes. The present results raise the possibility that in environments where multiple risks are common, targeted screening and training of neuropsychological functions could be of potential intervention value. If future causal research supports this hypothesis, for children with delays in neuropsychological development, providing activities that engage VMI, spatial awareness, or complex movement sequences might prove more directly effective in strengthening the cognitive foundations that support learning than providing nutritional supplementation alone.

Moreover, in the present study, the direct association between nutritional status and academic achievement was not significant. This finding may reflect characteristics of the sample. Nutritional risk is relatively common in this group, meaning that its limited variance was insufficient to explain individual differences in academic achievement. Alternatively, the effects of nutrition may operate more indirectly, influencing academic achievement through long-term growth and developmental pathways rather than through concurrent physiological status. In contrast, VMI appears to serve as a more direct indicator of currently available cognitive processing efficiency, which could explain its consistent associations even in the presence of nutritional adversity.

### 4.1 Practical implications

The findings of this study, while preliminary and correlational, offer some speculative considerations for educational practice in resource-limited settings, pending replication with more robust measures.

If further validated, the B-G test could be used to screen children for VMI. This might help teachers identify earlier those who may face academic challenges. Theoretically, training specifically targeting VMI could be more effective than focusing solely on physical fitness or repetitive motor skills. If future causal studies confirm such a link, low-cost activities could be explored as potential additions to physical education curricula, including target games, tracing geometric patterns on the playground, and rhythmic movement games. To illustrate, teachers might draw simplified B-G test figures on the playground and ask children to hop along the lines while naming the figures, or place cards along a shuttle run path for children to collect and sort by content (e.g., Chinese radicals or odd/even numbers). Such low-cost, cognitively engaging activities could be integrated into physical education or free play, though their efficacy requires future experimental validation.

If future evidence supports the efficacy of such approaches, teacher training could also be strengthened. During training, educators can gain insight into the connection between motor competencies and cognitive development while learning to design simple games. These activities require minimal equipment and can even utilize natural materials as substitutes. This approach enhances the enjoyment of play while making these activities more sustainable for long-term implementation in schools with limited resources.

### 4.2 Limitations

This study has several limitations.

The cross-sectional design precludes causal inferences. Future research could employ longitudinal designs or intervention trials to test whether improvements in VMI directly contribute to better academic achievement.

The measurement of SMI has limitations. Whilst our theoretical model proposes SMI, our primary research primarily assessed VMI, covering only a subset of sensorimotor function. It does not capture other dimensions such as proprioceptive processing, dynamic balance, or cross-modal integration. Future studies should use multifaceted measures of sensorimotor skills to identify which specific components are most strongly associated with academic achievement.

The modified five-figure version of the B-G test used in this study, while demonstrating acceptable psychometric properties, was not validated against the full nine-figure version. This limits the comparability of our findings with the extensive literature that has used the full B-G test and means that our results should be interpreted as providing preliminary evidence rather than definitive conclusions about the role of VMI in academic achievement. Future research must validate the abbreviated version against the full test, ideally in a similar population, before firm conclusions can be drawn. Moreover, the abbreviated test may not fully represent the complexity of visual-motor integration as originally conceptualized. The omission of four figures could have reduced the test's sensitivity to detect subtle deficits in VMI. Therefore, replication using the full B-G test is urgently needed.

Additionally, the subgroup analysis based on nutritional status involved a small malnourished sample (*n* = 26), limiting statistical power; these exploratory findings require replication. Furthermore, although we included grade as a covariate to control for developmental differences in motor performance, this may have overcontrolled variance, as academic scores were already grade-standardized. The sensitivity analysis without grade (Section 3.6) indicated that the speed–achievement association was not robust, suggesting that grade-related maturational differences may have contributed to the observed effects. Future studies should employ age-standardized motor measures or multilevel models to address this issue.

The assessment of motor competencies could be made more comprehensive. Future studies could include additional indicators such as aerobic endurance and muscular strength. In particular, motor skill assessments could incorporate both initial learning phases and consolidated performance, allowing for a more nuanced understanding of how different stages of motor skill acquisition relate to academic achievement.

Future research could include demographic variables related to family structure and emotional factors to better characterize the environmental context. It would also be valuable to explore the generalizability of these findings across children from different cultural backgrounds and economic settings.

## 5 Conclusion

This study provided preliminary correlational evidence consistent with a cognitive demand hierarchical model of motor competence in relation to academic achievement among children in resource-limited settings. VMI, which requires substantial cognitive engagement, appears to be the most consistent and domain-general correlate of academic success. In contrast, automated motor skills showed no association with academic achievement, and basic speed did not exhibit an independent relationship after accounting for grade-related maturational differences. These findings suggest that under resource-limited conditions, VMI, which requires sustained cognitive resources, appears to be more strongly associated with academic progress than general physical fitness or well-practiced motor skills. Future longitudinal studies are needed to establish causality, and replication using the full B-G test is also required. Researchers may further examine whether VMI-based interventions can help at-risk children through controlled experiments.

## Data Availability

The raw data supporting the conclusions of this article will be made available by the authors, without undue reservation.
