# Supplementary material for: Hierarchical motor competencies and academic achievement: visual-motor integration as the key correlate for school-age children in a disadvantaged context
Source: Front Psychol. 2026 Jun 11;17:1829790. doi: 10.3389/fpsyg.2026.1829790 (PMC13294086; doi:10.3389/fpsyg.2026.1829790)
Supplement: Supplementary file 2 [file Table_2.docx]

**Supplementary Material Table S2. Bivariate Correlations Among Standardized Motor Competency and Academic Achievement Variables (*n* = 155)**

| **Variable (z-score)** | **1** | **2** | **3** | **4** | **5** | **6** | **7** | **8** | **9** | **10** |
| --- | --- | --- | --- | --- | --- | --- | --- | --- | --- | --- |
| **1. BMI** |  |  |  |  |  |  |  |  |  |  |
| **2. Speed composite** | 0.135 |  |  |  |  |  |  |  |  |  |
| **3. Dribbling composite** | -0.099 | 0.360^**^ |  |  |  |  |  |  |  |  |
| **4. B-G test** | -0.059 | -0.207^**^ | -0.259^**^ |  |  |  |  |  |  |  |
| **5. Chinese language** | 0.047 | -0.087 | -0.008 | 0.348^**^ |  |  |  |  |  |  |
| **6. Mathematics** | 0.085 | -0.07 | -0.006 | 0.336^**^ | 0.728^**^ |  |  |  |  |  |
| **7. Academic index** | 0.036 | -0.08 | -0.014 | 0.375^**^ | 0.922^**^ | 0.899^**^ |  |  |  |  |
| **8. 30-m sprint** | 0.116 | 0.854^**^ | 0.238^**^ | -0.251^**^ | -0.022 | -0.009 | -0.015 |  |  |  |
| **9. 3×10-m shuttle run** | 0.115 | 0.854^**^ | 0.377^**^ | -0.102 | -0.126 | -0.111 | -0.122 | 0.46** |  |  |
| **10.Hand dribbling (dominant hand）** | -0.110 | 0.331^**^ | 0.989^**^ | -0.267^**^ | -0.01 | -0.025 | -0.026 | 0.214** | 0.352** |  |
| **11.Hand dribbling (non-dominant hand）** | -0.860 | 0.380^**^ | 0.989^**^ | -0.246^**^ | -0.005 | 0.013 | -0.002 | 0.256** | 0.393** | 0.956** |
