# Supplementary material for: Hierarchical motor competencies and academic achievement: visual-motor integration as the key correlate for school-age children in a disadvantaged context
Source: Front Psychol. 2026 Jun 11;17:1829790. doi: 10.3389/fpsyg.2026.1829790 (PMC13294086; doi:10.3389/fpsyg.2026.1829790)
Supplement: Supplementary file 3 [file Table_3.docx]

**Supplementary Material Table 3. Sensitivity Analyses: Independent Associations with Academic Achievement**

| **Variable** | **Clean Sample (*n* = 137)** | | **Full Sample (*n* = 156)** | | **Core Subjects Only**  **(*n* = 155)** | |
| --- | --- | --- | --- | --- | --- | --- |
|  | *β* | *p* | *β* | *p* | *β* | *p* |
| B-G test | 0.384 | <0 .001 | 0.436 | <0 .001 | 0.437 | <0 .001 |
| Speed composite | -0.333 | 0.039 | -0.345 | 0.019 | -0.343 | 0.039 |
| Dribbling composite | -0.159 | 0.366 | -0.008 | 0.96 | 0.021 | 0.894 |
