# Supplementary material for: Hierarchical motor competencies and academic achievement: visual-motor integration as the key correlate for school-age children in a disadvantaged context
Source: Front Psychol. 2026 Jun 11;17:1829790. doi: 10.3389/fpsyg.2026.1829790 (PMC13294086; doi:10.3389/fpsyg.2026.1829790)
Supplement: Supplementary file 4 [file Table_4.docx]

| **Variable** | **Model 1** | | **Model 2** | | **Collinearity** | |
| --- | --- | --- | --- | --- | --- | --- |
|  | *B (SE)* | *β* | *B (SE)* | *β* | | VIF |
| **Step 1: Control Variables** | |  |  |  | |  |
| Gender (male) | -0.084 (0.145) | -0.047 | 0.04 (0.144) | 0.022 | | 1.14 |
| BMI (z-score) | 0.016 (0.035) | 0.036 | 0.033 (0.033) | 0.077 | | 1.05 |
| **Step 2: Motor Competencies** | |  |  |  | |  |
| Speed composite |  |  | -0.045 (0.089) | -0.043 | | 1.29 |
| Dribbling composite |  |  | 0.105 (0.089) | 0.117 | | 1.244 |
| B-G test |  |  | 0.36(0.072) | 0.404^***^ | | 1.16 |
| **Model Summary** |  |  |  |  | |  |
| *R²* |  | 0.004 |  | 0.155 | |  |
| Adjusted *R²* |  | -0.01 |  | 0.127 | |  |
| *ΔR²* |  | 0.004 |  | 0.152 | |  |
| *F* for *ΔR²* |  | 0.267 |  | 8.91^***^ | |  |

**Supplementary Table S4. Hierarchical Regression Without Grade Covariate (*n* = 155)**

****p <0.001.*
